# Innate Immune Activation by Tissue Injury and Cell Death in the Setting of Hematopoietic Stem Cell Transplantation

**DOI:** 10.3389/fimmu.2015.00101

**Published:** 2015-03-16

**Authors:** Todd V. Brennan, Victoria R. Rendell, Yiping Yang

**Affiliations:** ^1^Department of Surgery, Duke University, Durham, NC, USA; ^2^Department of Medicine, Duke University, Durham, NC, USA; ^3^Department of Immunology, Duke University, Durham, NC, USA

**Keywords:** endogenous innate immune, graft-versus-host disease, hematopoietic stem cell transplantation, tissue injury

## Abstract

Allogeneic hematopoietic stem cell transplantation (Allo-HSCT) with donor lymphocyte infusion is the mainstay of treatment for many types of hematological malignancies, but the therapeutic effect and prevention of relapse is complicated by donor T-cell recognition and attack of host tissue in a process known as graft-versus-host disease (GvHD). Cytotoxic myeloablative conditioning regimens used prior to Allo-HSCT result in the release of endogenous innate immune activators that are increasingly recognized for their role in creating a pro-inflammatory milieu. This increased inflammatory state promotes allogeneic T-cell activation and the induction and perpetuation of GvHD. Here, we review the processes of cellular response to injury and cell death that are relevant following Allo-HSCT and present the current evidence for a causative role of a variety of endogenous innate immune activators in the mediation of sterile inflammation following Allo-HSCT. Finally, we discuss the potential therapeutic strategies that target the endogenous pathways of innate immune activation to decrease the incidence and severity of GvHD following Allo-HSCT.

## Introduction

Allogeneic hematopoietic stem cell transplantation (Allo-HSCT) with donor lymphocyte infusion is currently the treatment of choice for several types of hematological malignancies associated with high rates of relapse, including chronic myeloid leukemia (CML) in adults and acute myeloid leukemia (AML) and acute lymphoid leukemia (ALL) in children and adults (1). While donor lymphocytes are critical for the prevention of tumor relapse, they also cause graft-versus-host disease (GvHD), the major complication of Allo-HSCT (2). Clinical manifestations of acute GvHD include rash, diarrhea, gastrointestinal hemorrhage, and jaundice, which result from immune-mediated injury to the epithelial cells of the skin, gastrointestinal tract, and liver, respectively. In severe cases, GvHD can be fatal and is the leading cause of mortality of Allo-HSCT recipients.

**Figure 1:**
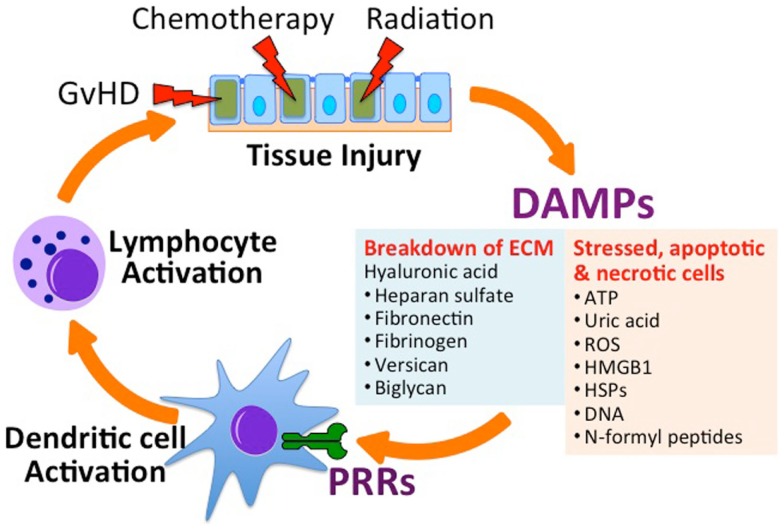
**Tissue injury in the setting of allogeneic hematopoietic stem cell transplantation (Allo-HSCT) promotes graft-versus-host disease (GvHD)**. Conditioning regimens used in HSCT, which includes total body irradiation and chemotherapy, lead to tissue injury and the subsequent release of danger-associated molecular patterns (DAMPs) from the extracellular matrix (ECM) and from stressed, apoptotic and necrotic cells. Acting on specific receptors on DAMPs activate host antigen presenting cells (e.g., dendritic cells) through specific pattern recognition receptors (PRRs), leading to the activation of donor-derived lymphocytes. Activated allo-specific lymphocytes can provoke further tissue injury from GvHD. ATP, adenosine triphosphate; ROS, reactive oxygen species; HMGB1, high-mobility group box 1 protein; HSP, heat shock protein.

Allogeneic hematopoietic stem cell transplantation requires myeloablation by total body irradiation (TBI) and/or chemotherapy in order to provide space in the hematopoietic compartment for the engraftment of donor hematopoietic stem cells. TBI and chemotherapy also provide lymphocyte suppression necessary to prevent immunological rejection of the Allo-HSCT graft, which can be mediated by even small amounts of host immune defense. These myeloablative “conditioning” regimens result in cellular damage and death that produce sterile inflammatory immune responses through the release of endogenous innate immune-stimulatory molecules referred to as damage-associated molecular patterns, or “DAMPs” (3–5) (Figure 1). Endogenous molecules such as uric acid (5), adenosine triphosphate (ATP) (6), deoxyribonucleic acid (DNA) (7), *N*-formyl peptides (NFPs) (8), and heparan sulfate (9) activate specific pattern recognition receptors (PRRs) on innate immune cells. An increasing number of PRRs have been identified; of these some of the best studied include toll-like receptors (TLRs), nucleotide-binding oligomerization domain (NOD)-like receptors (NLRs), the ATP receptor (P2X_7_R), and the formyl peptide receptors (FPR-1 and FPR-2). The downstream responses of these receptors on antigen presenting cells (APCs), such as dendritic cells (DCs), include increased expression of inflammatory cytokines and the upregulation of co-stimulation and antigen presenting molecules that play a critical role in initiating and orchestrating the resulting immune response.

Many PRRs play a dual role in the recognition of DAMPs and pathogen-associated molecular patterns, or “PAMPs,” derived from microbial infection such as lipopolysaccharide (LPS) from bacteria, single-stranded RNA from viruses, and unmethylated CpG DNA motifs from bacteria and viral sources (4, 10–13). Similar to DAMPs, PAMPs also contribute to the inflammatory immune response following HSCT. For example, the loss of GI tract integrity due to epithelial barrier damage from conditioning regimens prior to allo-HSCT, as well as from GvHD following allo-HSCT, allows the translocation of gut commensal bacteria into the systemic circulation (14). In addition, the immune-compromised HSCT recipient is at high risk for viral, bacterial, and fungal infections, all potent sources of PAMPs. A complete discussion of the activation of the innate immune system by PAMPs is outside the scope of this review, but has been reviewed by others (15). The relative contribution of DAMPs versus PAMPs to the inflammatory response in the setting of Allo-HSCT is currently unclear.

In the setting of Allo-HSCT, DAMPs released following tissue damage and cell death caused by cytotoxic conditioning regimens and GvHD have the potential to enhance immune activation and worsen GvHD. A better understanding of their role in promoting immune activation will aid in the design of effective strategies to improve the outcome of Allo-HSCT. Here, we briefly review the mechanisms of cell injury involved in conditioning regimens for Allo-HSCT, highlight the potential cellular and molecular mediators of sterile inflammation in the setting of HSCT, and discuss potential therapeutic strategies of regulating these pathways to control GvHD in the setting of Allo-HSCT.

## Tissue Injury from Conditioning Regimens

Multiple pathways of tissue injury occur following treatment with ionizing radiation (16, 17). Radioactive particles transfer their energy to tissues, resulting in the breaking of chemical bonds and the formation on unnatural chemical bonds. Radiation also creates free radicals and reactive oxidative species (ROS) that cause further direct tissue damage (18, 19). Following radiation, complex changes in cellular gene expression that vary between individuals, can lead to indirect cell damage (20). Tissues with high rates of proliferation, such as hematopoietic cells and enterocytes, are particularly susceptible to radiation injury as they become unable to meet their metabolic demands and unable to repair DNA damage prior to cell division (18).

Multiple chemotherapeutic regimens used in Allo-HSCT also injure host cells through a variety of cytotoxic mechanisms. While chemotherapeutic agents are dosed to be preferentially toxic to hematopoietic stem cells, they are capable of causing injury to most cell types (21). Efforts to decrease cell injury through the use of non-myeloablative conditioning regimens have been associated with decreased incidence and severity of GvHD compared to myeloablative regimen (22–24).

Cell responses to injury and stress following chemoradiation therapy include necrosis, apoptosis, and autophagy, all of which are pathways that can lead to cell death and subsequent innate immune activation (25). This response can be helpful in promoting engraftment of donor cells and in strengthening the alloimmune-dependent graft-versus-tumor (GvT) effect, or they can elicit detrimental effects by initiating GvHD. These responses to cell injury are described below.

## Responses to Cell Injury

### Necrosis

Necrosis is a cell death process involving cell membrane rupture that lacks the morphological and biochemical characteristics of autophagy or apoptosis. In the setting of HSCT, cell necrosis occurs following bone marrow myeloablation conditioning regimens and potentially from GvHD and infections resulting from immunosuppression. Although the initiating mechanisms are unclear, a characteristic set of changes occurs within the cell. There is a rapid loss of the cellular membrane ion potentials, cytoplasm, and cytoplasmic organelles swell, and there is a rise in cytosolic calcium levels, leading to protease and phospholipase activation. Mitochondrial dysfunction ensues and reactive oxygen species (ROS) are formed that further damage proteins, lipids, and DNA. Cell membrane integrity is eventually lost and membrane rupture occurs with release of intracellular contents into the extracellular space (26, 27). These released intracellular materials can act as DAMPs, alerting the innate immune system that an immune response is needed to either resolve a pathogen infection or initiate wound healing (28).

### Apoptosis

Apoptosis is a process of programed cell death involving morphological cell changes of pyknosis, karyorrhexis, cell membrane blebbing, and loss of cell surface adhesions (25, 29). Recipient hematopoietic and stromal cells undergo apoptosis following conditioning regimens used in HSCT, and T-cells undergo apoptosis during GvHD (30).

The uptake of apoptotic cells by APCs can be anti-inflammatory and even potentially tolerogenic (31, 32). Injection of apoptotic cells at the time of HSCT has been shown to improve engraftment and decrease allo-immunization (33, 34). In a murine model of Allo-GvHD, the infusion of apoptotic APCs prior to Allo-HSCT decreased GvHD severity and improved survival (35, 36). However, if not phagocytized in a timely fashion, apoptotic cells will undergo necrosis, exposing previously hidden DAMPs. For example, apoptotic cells from monocytes, macrophages, and multiple cell lines cause the production of inflammatory cytokines and chemokines (37–39), and increased apoptosis of recipient stromal cells is linked to more severe cases of acute GvHD (40). Overall, the pro- and anti-inflammatory properties of apoptotic cells in the setting of HSCT and GvHD merits further investigation.

### Autophagy

Cells under stress or undergoing starvation utilize autophagy to degrade and recycle organelles and intracellular macromolecules (41, 42). The role of autophagy in HSCT is unclear and is likely complex due to both pro- and anti-inflammatory properties of the autophagy process [reviewed by Leveque et al. (43)]. Treatment with radiation has been shown to upregulate autophagy in multiple cell types, particularly cells forming the gut barrier (44) and autophagy is theorized to limit inflammation by degrading inflammasomes (25, 45). In addition, the turnover of mitochondria by autophagy (mitophagy) is critical for removing damaged mitochondria that can be the source of multiple DAMPs (46–48).

## Sources of Endogenous Innate Immune Activators

A variety of endogenous innate immune activators, or DAMPs, released during the cell death process have been identified. The following section will review the role of DAMPs in contributing to APC activation in the setting of HSCT, leading to GvHD.

### Mitochondria

**Figure 2:**
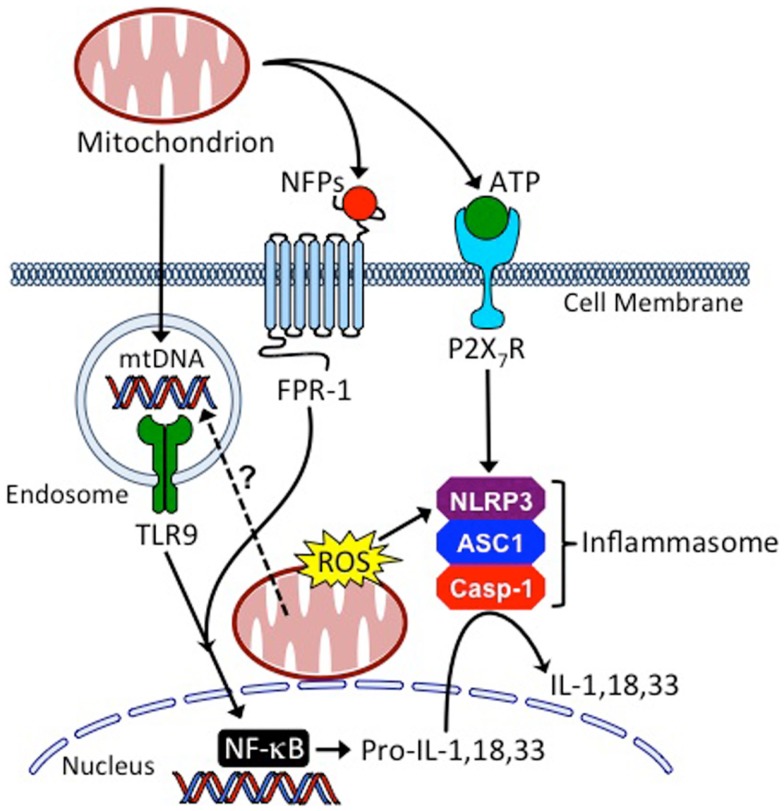
**Mitochondria are central regulators of innate inflammatory responses**. Cells injured from conditioning regimens used in HSCT, or as the result of GvHD, can leak mitochondria into the extracellular space where they release mitochondrial DNA (mtDNA), *N*-formyl peptides (NFPs), and adenosine triphosphate (ATP), causing host APC activation through binding to toll-like receptor 9 (TLR9), formyl peptide receptor 1 (FPR1) and the leukocyte purinergic receptor (P2X_7_R), respectively. Additionally, mitochondria of injured cells release reactive oxygen species (ROS) and their mtDNA may enter the endo-lysosomal compartment through the process of mitophagy to activate TLR9. These processes coordinate the production and maturation of inflammatory cytokines, such as interleukin (IL)-1, IL-18, and IL-33, through the activation of NF-κB (nuclear factor kappa-light-chain-enhancer of activated B cells) and the NLRP3 (NOD-, LRR-, and pyrin domain-containing 3 inflammasome). ASC1, apoptosis-associated speck-like protein containing a CARD; Casp-1, caspase-1.

Mitochondria are a rich endogenous source of DAMPs, and there is increasing evidence that mitochondria play a central role in innate immune activation leading to regulation of the adaptive immune response (49–52) (Figure 2). Mitochondria evolved from an endosymbiotic relationship between eukaryotic cells and bacteria and retain structural features common to bacteria (53). Of particular relevance to their immune stimulating potential are their NFP-containing proteins and their circular DNA genome featuring unmethylated CpG motifs that resemble DNA of bacterial or viral origin. This is in contrast to mammalian genomic DNA, where between 60 and 90% of all CpG repeats are methylated (54).

There is growing evidence that mitochondrial DNA (mtDNA) is released in the setting of tissue injury. For example, plasma mtDNA is elevated in trauma patients (55), in patients with femur fracture (56), and in patients with acute liver injury (57–59). The release of mtDNA from injured tissue and cells may also play a key role in the pathogenesis of autoimmune diseases such as systemic lupus erythematosus (SLE) and rheumatoid arthritis (RA) (60–63).

Specific oligodeoxynucleotides (ODNs) derived from mtDNA have been identified that activate DCs *in vitro* based on their content of unmethylated CpG motifs (64). CpG ODNs are recognized by TLR9, leading to MyD88 activation and either an interferon (IFN) regulatory factor 3 (IRF3)- and IRF7-dependent type-1 IFN response or an NF-κB (nuclear factor kappa-light-chain-enhancer of activated B cells)-initiated inflammatory response, depending on whether they localize to the endosomal or lysosomal compartment, respectively (65–68). In addition, mtDNA contains a high percentage of 8-hydroxy-2′-deoxyguanosine (8-OHG) residues that make mtDNA resistant to DNAses and increase their inflammatory potential (48, 69, 70). The high content of 8-OHG is the result of leaky oxidative machinery, the lack of efficient DNA repair mechanisms, and the absence of protective histones.

The administration of CpG ODNs at the time of Allo-HSCT accelerates GvHD in a host APC-TLR9-dependent manner and a host IFN-γ-dependent manner, but independent of host IL-6, IL-12, or natural killer (NK) cells (71). Interestingly, CpG administration also increased bone marrow rejection in a manner dependent on donor APC-TLR9 activation. In an MHC-mismatch murine model of HSCT using TLR9^−/−^ recipient mice, the GvHD clinical score of TLR9^−/−^ mice was significantly lower than that of wild-type mice, while no significant differences were observed when weight loss was considered alone (72).

Another GvHD study examining the role of MyD88, TRIF, TLR2/4, and TLR9 found that while deficiency in all these molecules decreased the intestinal immunopathology of GvHD, only TLR9 deficiency improved survival (73). Deficiencies in MyD88 and TLR9 also reduced the number of apoptotic cells in the gut in the same model of intestinal GvHD. In support of these findings, the administration of an inhibitory ODN that blocks TLR9 activation was shown to decrease severity of intestinal GvHD, measured by reduction in caspase-3 staining and decreased apoptotic cell counts (73). These results suggest a role of TLR9 activation by unmethylated CpG containing DNA, an endogenous source of which is mtDNA, in the inflammatory pathology of GvHD.

Mitochondrial-encoded proteins are initiated with *N*-formylmethionine residues similar to bacterial proteins (74). NFPs-derived from mitochondrial proteins are recognized by the formyl peptide receptors, FPR1 and FPR2, with high- and low-affinity, respectively. FPR1 and FPR2 are seven-transmembrane domain, G protein-coupled receptors, and their activation leads to neutrophil calcium mobilization, p38 and Erk1/2 activation, degranulation, oxidative burst, and chemotaxis (51). The role of NFPs in GvHD is unknown, but intravascular gradients of NFPs have been shown to guide neutrophils to sites of liver injury in mice (75).

Mitochondria also produce ATP, the main source of cellular energy, through oxidative phosphorylation. At high-extracellular concentrations, ATP activates the cell surface purinergic receptor, P2X_7_R, leading to activation of the NLRP3 inflammasome (6, 76, 77). Extracellular ATP has recently been shown to have a role in GvHD (78). Following TBI and during the development of acute GvHD, ATP level have been observed to be elevated in the peritoneal fluid of mice and humans, and GvHD survival in mice was improved by the breakdown of ATP with apyrase, with ATP receptor antagonism, and in mice genetically deficient for the P2X_7_ receptor (78).

Mitochondrial activation of the NLRP3 inflammasome also occurs through the release of ROS, resulting in the production of IL-1β and IL-18 through caspase-1 activation (79). ROS released from neutrophils at the site of gastrointestinal tract injury following radiation has been demonstrated. In irradiated mice, neutrophils strongly upregulate inducible phagocyte NADPH oxidase-1 (NOX1) (80). Deficiency in *Cybb*, the gene coding for NOX2, impairs ROS production, and irradiated *Cybb*-deficient allo-HSCT mouse recipients had protection from GVHD and improved survival (81).

In summary, as may be predicted from their bacterial origins, mitochondrial-derived molecules are capable of activating multiple inflammatory pathways used in host defense against microbial pathogens. There is increasing evidence that mitochondria are potent endogenous innate immune activators that can promote the pathogenesis of GvHD following Allo-HSCT.

### Extracellular matrix

Molecules from the extracellular matrix (ECM) also have been shown to become pro-inflammatory when they are fragmented and/or released. A number have been identified as having a role in immune activation including heparan sulfate, fibronectin, fibrinogen hyaluronate, biglycan, and tenascin (9, 82–88).

Hyaluronate and heparan sulfate induce DC maturation and alloimmune activation following recognition by TLR4 (9, 83). In a murine model examining skin manifestations of acute GvHD, hyaluronate interactions with CD44 promotes lymphocyte adherence to recipient endothelium (89). Serum heparan sulfate levels are significantly elevated in the setting of acute GvHD in a mouse model of Allo-HSCT (90). Increased serum heparan sulfate levels result in increased donor T-cell activation and proliferation along with GvHD severity. In human recipients of Allo-HSCT, similar elevations in serum heparan sulfate are detected and correlate with increasing GvHD grade (90).

### High-mobility group box protein 1

High-mobility group box protein 1 (HMGB1) is a non-histone nuclear protein whose role is to bind DNA and regulate transcription. HMGB1 has been implicated in causing inflammation associated with ischemia–reperfusion injury, acute respiratory distress syndrome, multi-organ distress syndrome, trauma, and autoimmunity [reviewed by Bianchi et al. (91)]. Serum levels of HMGB1 are elevated in patients who developed acute GvHD following Allo-HSCT (92). In Allo-HSCT recipients conditioned with myeloablation, there is an association between polymorphisms in HMGB1 genotype and outcomes after Allo-HSCT (93). Interestingly, thrombomodulin, a natural neutralizer of HMGB1, has been demonstrated to treat refractory acute GvHD complicated by thrombotic microangiopathy (94).

### Monosodium urate crystals

In a study identifying the immune-stimulatory products of cell damage released following treatment with ultraviolet (UV) radiation, uric acid was identified as a danger signal that causes priming of CD8^+^ T-cell responses. When added to DCs *in vitro*, uric acid rapidly upregulates expression of costimulatory molecules (5). Monosodium urate (MSU) is among several types of crystalline molecules that have been shown to initiate inflammatory immune responses through the activation of NLRs, including asbestos, silica, heme, and heme-like molecules (95–98). MSU crystals are potent activators of the NALP3 inflammasome, leading to release of IL-1β and IL-18, which act downstream to stimulate parenchymal cells to secrete chemokines and promote neutrophil infiltration (4, 99). In a mouse model of HSCT, uric acid levels in the peritoneal cavity were higher post TBI and chemotherapy pre-conditioning regimens. When uric acid depletion was induced with early uricase administration, treated mice had improved survival and lower IL-1β levels compared to control mice (100). A phase I study further explored the role of uric acid in GvHD by treating patients with recombinant urate oxidase for 5 days during their conditioning regimen for HSCT. The patients treated with urate oxidase indeed had lower serum levels of uric acid compared to matched controls, and, post-HSCT, these patients had a significantly decreased incidence of acute GvHD (101). A contradictory finding was shown in a retrospective study of 228 patients where low levels of uric acid at the time of HSCT was associated with significantly lower severity of GvHD (102). It was postulated that the antioxidant capacities of uric acid are also important for the prevention of GvHD and could outweigh its pro-inflammatory effects in some cases. Further investigation is needed to understand the competing roles of uric acid as an inflammatory mediator and an antioxidant and its contribution to GvHD pathogenesis.

### Heat shock proteins

Heat shock proteins (HSPs) are molecular chaperones with a role in the formation of tertiary and quaternary protein structures and translocation within the cell. Numerous studies have reported on the immune-stimulatory potential of HSPs, which can elicit both innate and adaptive immune responses. In a human skin explant model, a high correlation was shown between the grade of GvHD and level of Hsp70 (103). In rats, Hsp70 expression in the spleen and lymph nodes is increased in the setting of GvHD (104). In human patients, homozygous Hsp70 gene polymorphisms are associated with a higher incidence and severity of GvHD (105).

## Therapeutic Potential

Current therapeutic strategies for the prevention and management of acute GvHD following allogeneic HSCT rely on immunosuppressive medications such as corticosteroids, calcineurin inhibitors, mTOR inhibitors (e.g., sirolimus), and antimetabolites such as methotrexate and mycophenolate (106). Despite these aggressive approaches, GvHD is the principal cause of mortality among bone marrow transplant recipients. Additionally, the global immunosuppressive nature of these medications interferes with the beneficial properties of donor lymphocytes, such as the GvT response necessary for the prevention of tumor relapse (107) and immune reconstitution necessary for protection against microbial infections (108). Addressing inflammation derived from the endogenous innate immune agonists summarized above may provide mechanism-based treatments for GvHD.

**Figure 3:**
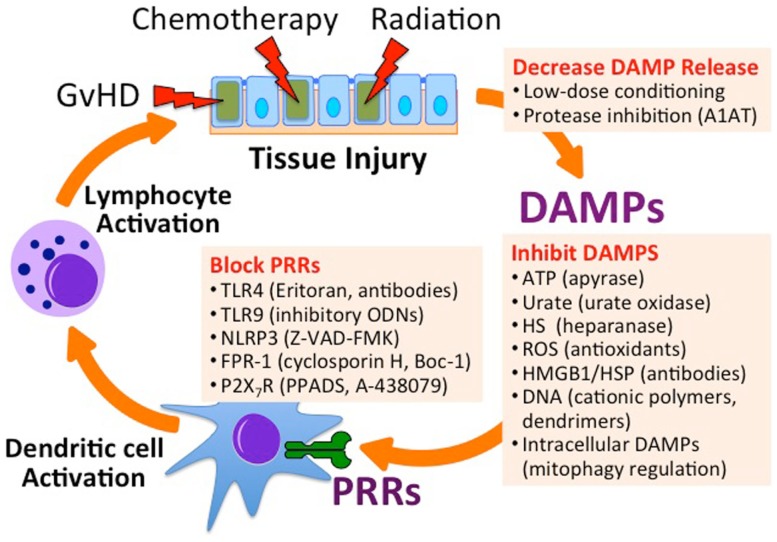
**Inhibiting endogenous innate immune activation in the setting of Allo-HSCT**. Utilizing low-dose chemoradiation conditioning regimens and protease inhibitors can minimize the release of DAMPs. Specific DAMPs can be targeted for degradation, inhibited by binders, or targeted for degradation. Antagonists can inhibit PRRs that recognize DAMPs. A1AT, alpha-1 antitrypsin; ODN, oligodeoxynucleotide; Z-VAD-FMK, carbobenzoxy-valyl-alanyl-aspartyl-[*O*-methyl]-fluoromethylketone; Boc-1, tert-butyloxycarbonyl-methionyl-leucyl-phenylalanine; PPADS, pyridoxal-phosphate-6-azophenyl-2′,4′-disulfonic acid; A-438079, 3-((5-(2,3-dichlorophenyl)-1H-tetrazol-1-yl)methyl pyridine).

One method to reduce endogenous inflammation derived from the release of DAMPs from injured, necrotic, or apoptotic cells is to reduce the severity of myeloablative conditioning before Allo-HSCT (23, 24) (Figure 3). A balance must be obtained between minimizing conditioning therapy and preventing rejection of the stem cell graft by host lymphocytes. One method for improving stem cell engraftment utilizes donor NK cells to aide in the elimination of host hematopoietic cells and augment the GvT response (109, 110). For this purpose, donor NK cell infusion is performed at the time of Allo-HSCT or cord blood transplantation in clinical trials for the treatment of hematologic malignancies (ClinicalTrials.gov Identifier: NCT01807611, NCT01621477, and NCT01619761). In this setting, donor NK function can be augmented using synthetic TLR9 agonists (e.g., CpG DNA), TLR3 agonists [e.g., Poly(I:C)], or IL-2 therapy.

In addition to minimizing myeloablative pre-conditioning toxicity, there are a number of strategies focused on targeting specific DAMPs and subsequent innate immune activation. Dying and injured cells release ATP that activates its receptor, P2X_7_R, on innate immune cells contributing to inflammatory response to HSCT pre-conditioning therapy and GvHD (78). Wilhelm and colleagues have shown that metabolizing extracellular ATP with apyrase or P2X_7_R antagonism with pyridoxal-phosphate-6-azophenyl-2′,4′-disulfonic acid (PPADS) is protective against GvHD in a murine model of allo-HSCT (78). As the authors also showed that ATP levels in the peritoneal cavity of human patients with GvHD following HSCT are dramatically elevated, methods of preventing the pathway of endogenous innate immune activation seem therapeutically promising.

Endogenous DNA, particularly hypomethylated CpG-rich mtDNA, is a TLR9-dependent innate immune activator, and methods to absorb extracellular DNA or blocking TLR9 are both potential avenues for decreasing GvHD severity. For example, using the synthetic TLR9 receptor inhibitory oligonucleotide, ODN-2088, Heimesaat and colleagues were able to decrease intestinal manifestation of GvHD in an irradiation-independent murine Allo-HSCT model (73). In a CpG-dependent model of acute liver failure, Lee and colleagues were able to use DNA binding polymers *in vivo* to prevent CpG related mortality in mice (111). Using these polymers at the time of either irradiation- or chemotherapy-based conditioning for Allo-HSCT, or at the onset of GvHD, has the potential to dampen the GvHD-immune response, but has not yet been tested.

Chemotherapy, irradiation, and GvHD result in tissue injury that can also lead to the degradation of ECM and the release of inflammatory glycosaminoglycans (GAGs), such as hyaluronate and heparan sulfate. GAGs can be released directly by glycolytic enzymes (e.g., heparanase), or by the proteolysis of extracellular or membrane bound proteoglycans to which they are attached. Alpha-1 antitrypsin (A1AT) is an abundant serum serine protease inhibitor critical for the prevention of neutrophil elastase-induced lung injury in the setting of chronic inflammation (112). The general immunosuppressive properties of A1AT have been observed in the prevention of acute myocardial ischemia–reperfusion injury (113) and ischemia–reperfusion-induced lung injury (114). A1AT has also been shown to prolong islet allograft survival in mice (115, 116). We and others have shown that administration of human A1AT decreases GvHD in murine models of Allo-HSCT (90, 117, 118), and it has recently been shown to preserve and enhance the NK-mediated GvT effect (119). A1AT is currently being studied in clinical trials for the treatment of steroid refractory GvHD (ClinicalTrials.gov Identifier: NCT01700036 and NCT01523821). Alternatively, the receptor for heparan sulfate, TLR4 (120), can be blocked by TLR4 antagonist antibodies (121) or TLR4 inhibitors such as Eritoran (122), TAK-242 (123), Ibudilast (124), and glucosamine dendrimers (125).

## Future Perspectives

The pathogenesis of GvHD following Allo-HSCT is complex and multifactorial, resulting in significant obstacles for progress in this area of research. Successful attempts to address this critical problem for Allo-HSCT patients have focused on decreasing cytotoxic effects of chemoradiation pre-conditioning, decreasing microbial translocation across the damaged GI tract, and, more recently, targeting PRRs for both endogenous and exogenous sources of innate immune activation.

The potential for targeting DAMPs released following cell injury is appealing given the desire to directly target GvHD pathophysiology and minimize attenuation of the GvT effects. Intriguing future directions involve targeting all aspects of endogenous innate immune activation, including blocking the release of DAMPs, binding and inactivating extracellular DAMPs, blocking or inhibiting PRRs that recognize DAMPs, and blocking intracellular signal transduction molecules downstream of PRRs (Figure 3). As the role of intracellular DAMPs such as mitochondria and cytoplasmic DNA in the inflammatory response is being revealed, new insight into the pathogenesis of GvHD may be illuminated as well.

In summary, there is increasing evidence that innate immune activators released from damaged tissue potentiate GvHD and that targeting these endogenous activators and their receptors is a promising methodology for controlling GvHD while maintaining a competent immune system.

## Conflict of Interest Statement

The authors declare that the research was conducted in the absence of any commercial or financial relationships that could be construed as a potential conflict of interest.
